# The Self-Disgust Scale Revised Version: validation and relationships with eating disorder symptomatology

**DOI:** 10.1186/2050-2974-2-S1-O48

**Published:** 2014-11-24

**Authors:** Jessica Moncrieff-Boyd, Karina Allen, Sue Byrne, Kenneth Nunn

**Affiliations:** 1 University of Western Australia, Perth, Australia; 2 The Children's Hospital at Westmead, Sydney, Australia

## 

Disgust has been recognised as a significant emotion in Anorexia and Bulimia Nervosa. Self-disgust, a discrete emotional experience characterised by feelings of revulsion and abhorrence at the self, has gained recent empirical attention among psychological conditions such as Major Depressive Disorder, but has yet to be explored in eating disorders. To date, only one self-report measure of self-disgust has been developed. This presentation will address the validation of a revised version of the Self-Disgust Scale (Overton, Markland, Taggart,Bagshaw and Simpson, 2008), which was modified in order to capture self-disgust as a distinct construct assessing visceral qualities of repulsion and abhorrence at the body and self, as well as low levels of self-acceptance. The revised version of the scale was administered to 746 university undergraduates (64% female) and associations were examined between scores on the revised Self-Disgust Scale and scores on the Eating Disorder Examination Questionnaire. Results revealed a significant, positive relationship between levels of self-disgust and eating disorder symptomatology, including global symptom scores and the presence of specific eating disorder behaviours. These findings will be discussed in regard to their wider implications for a potential role of disgust at the self and the body in eating disorder phenomenology.

This abstract was presented in the **Assessment** stream of the 2014 ANZAED Conference.

